# Breaking new ground: challenging existing asthma guidelines

**DOI:** 10.1186/1471-2466-6-S1-S6

**Published:** 2006-11-30

**Authors:** David Price, Mike Thomas

**Affiliations:** 1 Department of General Practice and Primary Care, University of Aberdeen, Foresterhill Health Centre, Westburn Road, Aberdeen AB25 2AY, UK

## Abstract

**Background:**

While we have international guidelines and various national guidelines for asthma diagnosis and management, asthma remains poorly controlled in many children and adults. In this paper we review the limitations of current asthma guidelines and describe important issues and remaining questions regarding asthma guidelines for use, particularly in primary care.

**Discussion:**

Clinical practice guidelines based on evidence from randomized controlled trials are considered the most rigorous and accurate. Current evidence-based guidelines are written predominantly from the perspective of the patient with a clear-cut asthma diagnosis, however, and tend not to consider the heterogeneity of asthma or to accommodate individual patient variations in response to treatment or their needs, differences in practice settings, or local differences in availability and cost of therapies. The results of randomized controlled trials, which are designed to establish efficacy of treatment under ideal conditions, may not apply to 'real-world' clinical practice, where patients are unselected, monitoring is less frequent, and effectiveness – the benefit of treatment in routine clinical practice – is the most relevant outcome. Moreover, most guidelines see asthma in isolation rather than considering other factors that may impact on asthma and response to asthma therapy, particularly age, allergic rhinitis, cigarette smoking, adherence, and genetic factors. When these links are recognized, guidelines rarely provide practical recommendations for treatment in these scenarios. Finally, there is some evidence that general practitioners are not convinced of the applicability of asthma guidelines to their practice settings, especially when those writing the guidelines principally work in specialist practice.

**Conclusion:**

Developing country-specific guidelines or, ideally, local guidelines could provide more practical solutions for asthma care and could account for regional factors that influence patient choice and adherence to therapy. Pragmatic clinical trials and well-designed observational trials are needed in addition to randomized controlled trials to assess real-world effectiveness of therapies, and such evidence needs also to be considered by guideline writers. Finally, practical tools to facilitate the diagnosis and assessment of asthma and factors responsible for poor control, such as associated allergic rhinitis, limited adherence, and smoking behavior, are needed to supplement treatment information provided in clinical practice guidelines for asthma.

## Background

Asthma is estimated to affect 300 million people worldwide, with an expected increase to 400 million worldwide by 2025 [1,2]. A chronic inflammatory disease of the airways, asthma causes 0.25 million deaths annually and substantial socioeconomic burden around the globe [1,2]. Moreover, despite the development and dissemination in recent years of both international guidelines and various national guidelines for asthma diagnosis and management, there is evidence that asthma is frequently not well controlled in many children and adults [3-5]. In this paper we review the limitations of current asthma guidelines and describe important issues and remaining questions regarding asthma guidelines for use in primary care practice.

## Overview of current guidelines

The systematic establishment of a thorough evidence base is currently considered the most rigorous and accurate means to develop clinical practice guidelines [6,7]. Developing evidence-based consensus guidelines is an enormous undertaking in a field such as asthma, involving a major literature search, consideration of thousands of papers, and time commitment by numerous individuals in the respiratory medical community. Several hierarchies of evidence and grading recommendations have been used by different working groups [6-8]; those used by the Scottish Intercollegiate Guidelines Network are presented in Table 1. The Grades of Recommendation Assessment, Development and Evaluation Working Group has recently proposed a system for grading quality of evidence and strength of recommendations that can be applied across a wide range of interventions and contexts [7].

**Table 1:** Hierarchy of levels of evidence from published papers, and grades of recommendation based on this hierarchy, as used by the Scottish Intercollegiate Guidelines Network [6]

Hierarchy of levels of evidence from published papers	
1++	High quality meta-analyses, systematic reviews of randomized controlled trials (RCTs), or RCTs with a very low risk of bias
1+	Well conducted meta-analyses, systematic reviews of RCTs, or RCTs with a low risk of bias
1-	Meta-analyses, systematic reviews of RCTs, or RCTs with a high risk of bias
2++	High quality systematic reviews of case control or cohort studies
	High quality case control or cohort studies with a very low risk of confounding, bias, or chance and a high probability that the relationship is causal
2+	Well conducted case control or cohort studies with a low risk of confounding, bias, or chance and a moderate probability that the relationship is causal
2-	Case control or cohort studies with a high risk of confounding, bias, or chance and a significant risk that the relationship is not causal

3	Non-analytic studies, eg, case reports, case series

4	Expert opinion
Grades of recommendation	
A	At least one meta-analysis, systematic review, or RCT rated as 1++ and directly applicable to the target population, *or*
	A systematic review of RCTs or a body of evidence consisting principally of studies rated as 1+, directly applicable to the target population and demonstrating overall consistency of results
B	A body of evidence including studies rated as 2++, directly applicable to the target population and demonstrating overall consistency of results, *or*
	Extrapolated evidence from studies rated as 1++ or 1+
C	A body of evidence including studies rated as 2+, directly applicable to the target population and demonstrating overall consistency of results, *or*
	Extrapolated evidence from studies rated as 2++
D	Evidence level 3 or 4, *or*
	Extrapolated evidence from studies rated as 2+

Reprinted with permission from the Scottish Intercollegiate Guidelines Network (SIGN) [6].

The Global Initiative for Asthma (GINA) was first launched in 1993 as a program to reduce asthma prevalence, morbidity, and mortality in collaboration with the World Health Organization and the National Heart, Lung, and Blood Institute of the National Institutes of Health in the United States. GINA has published evidence-based asthma guidelines since 2002 [9]; annual updates are available on the GINA website [10]. These guidelines emphasize the fact that asthma is a chronic inflammatory disorder of the airways and that, while asthma exacerbations are episodic, airway inflammation is chronically present [10]. Exposure to allergens is listed as a common risk factor. The fact that medication must be taken every day by most patients to control symptoms, to improve lung function, and to prevent attacks is noted. Criteria defining the control of asthma are presented in the guidelines (Table 2). Moreover, the guidelines provide criteria for determining asthma severity; and recommendations for pharmacological management of asthma are outlined according to a stepwise approach based on asthma severity.

**Table 2:** Asthma control as defined by the Global Initiative for Asthma guidelines [10]

Minimal (ideally no) chronic symptoms, including nocturnal symptoms
Minimal (infrequent) exacerbations
No emergency visits
Minimal (ideally no) need for p.r.n. (as-needed) β_2_-agonist
No limitations on activities, including exercise
PEF circadian variation of less than 20%
(Near) Normal PEF
Minimal (or no) adverse effects from medicine

PEF, peak expiratory flow.

Other examples of English-language, strict evidence-based guidelines are the British Asthma Guidelines, which were revised most recently in November 2005 and are available on the Scottish Intercollegiate Guidelines Network website [11], and the Canadian Consensus Asthma Guidelines [12], which are pragmatic, evidence-based guidelines based on the same hierarchy of evidence as the British guidelines and were last revised in 2003 [13,14]. The International Primary Care Airways Group more recently developed a handbook to guide primary care physicians in the management of chronic airways disease [15]. This handbook provides an algorithmic approach to differential diagnosis of allergic rhinitis, asthma, and chronic obstructive pulmonary disease using validated questionnaires and diagnostic guides that are consistent with evidence-based guidelines developed by GINA, by the Global Initiative for Chronic Obstructive Lung Disease, and by the Allergic Rhinitis and its Impact on Asthma (ARIA) initiative. In addition, guidelines for the diagnosis of respiratory disease and treatment of allergic rhinitis have been published by the International Primary Care Respiratory Group [16,17].

## Limitations of current guidelines

Clinical practice guidelines are by their nature general recommendations aimed for broad applicability in the clinical setting. Applicability, however, is limited by several factors. One of the challenges to the daily use of current asthma guidelines by physicians is that these guidelines tend to be disease-oriented, not patient-oriented. Guidelines are written not from the perspective of the patient who comes to the physician's office with symptoms, but rather from the perspective of the patient with a clear-cut asthma diagnosis. Symptom-based guidelines are needed [2]. In addition, there are patients who do not fit within many current guideline definitions; for example, children with intermittent wheezing that does not appear to be the classical asthmatic phenotype [18]. Moreover, guideline recommendations tend to be based on disease severity without accounting for concomitant conditions, such as allergic rhinitis, or the time course of disease – factors used by clinicians to individualize the diagnosis and the treatment plan.

In addition, clinical practice guidelines for asthma tend not to consider the heterogeneity of asthma or to accommodate individual patient variations in response to treatment or their needs, differences in practice settings, or local differences in availability and cost of therapies. Guideline recommendations are generally made on the basis of grouped mean data, and fail to recognize individual heterogeneity. For instance, a recent crossover trial comparing an inhaled corticosteroid with a leukotriene receptor antagonist for the treatment of persistent childhood asthma found that, while both treatments were effective, on average the effect was greater in those treated with inhaled corticosteroid. When individual patient responses on each treatment were compared, however, 29% of children had better asthma control (asthma control days/week) on the leukotriene receptor antagonist, indicating that for this subgroup the leukotriene receptor antagonist would be the ideal monotherapy [19].

Asthma is most commonly managed in the community in general practice settings, and the organization of medical care will affect how asthma care can be delivered. Differential diagnoses vary according to location, with infectious disease being more common in less developed countries. Moreover, currently available guidelines are based on the assumption that the recommended drugs are available and affordable [2], an assumption that is not true in many parts of the world. There is therefore a pressing need for local guidelines [8]. To be useful to primary care physicians, these guidelines must be in the local language as well as physically available, whether by Internet or in print.

### Limitations of the evidence-based approach

The results of well-designed randomized controlled trials (RCTs) and meta-analyses of RCTs are awarded the highest level of evidence in hierarchies of evidence-based data collection used to formulate clinical practice guidelines [6,8]. RCTs, however, are designed to maximize internal validity and to minimize confounding factors by studying a tightly defined population in a controlled setting, and therefore to establish the *efficacy *of treatment; namely, the benefit of the treatment under ideal conditions. RCTs are not primarily concerned with the external validity or generalizability of the findings to whole populations, and differences between the homogeneity of the trial population and the heterogeneity of the general population labeled as having asthma may limit the generalizability of RCT findings [20]. These trials generally enroll a carefully selected patient population meeting strict inclusion criteria and exclusion criteria, involve frequent clinical and laboratory monitoring, and measure objective parameters of efficacy. In 'real-world' clinical practice, however, patients are unselected, monitoring tends to be less frequent and less complete, and *effectiveness *– the benefit that treatment produces in routine clinical practice [21] – is the most relevant outcome.

Herland and coworkers [22] found that only 5.4% of outpatients with asthma recruited at 12 centers in their study would have qualified for enrollment in a classical RCT. If patients with symptoms or patients who regularly used inhaled corticosteroids were also excluded, as they are in some RCTs, the percentage fell to 3.3% of asthma outpatients. The patients entering RCTs are therefore not representative of asthma patients in primary care [22]. Moreover, the frequent clinical care and monitoring of patients in RCTs is not typical of 'real-world' clinical situations and thus the outcomes of the RCTs may not be attainable in 'real-world' clinical situations. Finally, measures of airway obstruction do not correlate with patient-reported measures, and it is therefore important to assess at least one endpoint from each of these categories in clinical trials [20,23,24].

Information gained from pragmatic trials and observational studies can be a valuable adjunct to that gained by RCTs. Pragmatic trials, which may be blinded or open label, are designed to more closely replicate conditions in clinical practice, including variability in patient characteristics and the use of a management protocol rather than a predetermined assigned treatment [21]. Observational studies are valuable to examine large groups of patients, to examine long-term outcomes, to examine rare but important outcomes such as mortality, and to examine outcomes that may not be easily assessed in RCTs, such as pharmacoeconomic data. Recent comparisons of results obtained by RCTs and by observational studies found that effects of treatment determined in observational studies were not systematically greater or qualitatively different from those of RCTs comparing the same treatments [25,26]. The reliance on RCTs as the highest level of evidence is therefore being challenged [27]; even regulatory agencies are now beginning to review evidence from well-designed observational research when making labeling evaluations. On the contrary, the quality of design and reporting of many observational studies has been questioned [28]. The same attention to design, the control of confounding factors, and complete reporting are clearly as necessary for observational studies as for RCTs.

Evidence-based guidelines are further limited by the time lag involved in the process, typically entailing 1–2 years, of reviewing the literature, arriving at a consensus, and actually writing and publishing the guidelines. This allows for potentially relevant studies to be published but not assessed. One mechanism for acknowledging more recent papers might be to provide electronic links to them from the guidelines, noting that the information they contain may be relevant but will not be commented on in the present guidelines.

### Lack of accounting for comorbid conditions and confounding factors

Most guidelines see asthma in isolation rather than considering other factors that may impact on asthma and the response to asthma therapy, particularly allergic rhinitis, cigarette smoking, adherence to therapy, and genetic factors.

The association between the upper airways and the lower airways has been recognized for two millennia: as long ago as 200 AD, Galen recommended purging the nostrils of secretions in order to relieve the lungs. In the late nineteenth century, Charles Blackley linked hay fever and asthma [29]. Current evidence for the many pathophysiological and epidemiological links between asthma and allergic rhinitis is discussed in other papers in the present supplement [30,31]. In recent clinical guidelines, however, with the exception of the ARIA guidelines [32], these links between allergic rhinitis and asthma are not fully addressed.

The GINA guidelines note that 'special considerations are required in managing asthma in relation to ... rhinitis'; however, there is no further guidance – such as a recommendation to examine the nose or to ask the patient whether or how allergic rhinitis worsens their asthma [10]. Moreover, the guidelines note that treatment of rhinitis may improve asthma symptoms, citing results of an observational study and a review [33,34]. They do not, however, cover the concept that asthma and allergic rhinitis are related conditions linked by one common airway. Therefore, while the GINA guidelines mention the association between asthma and allergic rhinitis, they fall short of describing practical recommendations for concomitant treatment. The British asthma guidelines and the Canadian asthma guidelines cited earlier are similarly lacking in providing advice on concomitant treatment.

The ARIA guidelines [32] were the first to stress the connection between allergic rhinitis and asthma. Since their publication in 2001 new data have been published that support these recommendations, as summarized in another paper in the present supplement [30]. The ARIA guidelines note that allergic rhinitis should be considered one of the risk factors for asthma [32]. Moreover, these guidelines recommend evaluating patients with persistent allergic rhinitis for asthma and evaluating patients with asthma for rhinitis. A 'combined strategy' to treat the upper airways and the lower airways is recommended [32].

The early evidence indicates that treating allergic rhinitis may help to control asthma symptoms. A recent post-hoc evaluation of the Clinical Observation of Montelukast as a Partner Agent for Complementary Therapy study showed the benefit of montelukast, a potent leukotriene receptor antagonist, for patients with coexisting asthma and rhinitis [35]. Cysteinyl leukotrienes are key mediators of both allergy and allergic rhinitis [32,36,37]; montelukast, which is approved to treat both asthma and allergic rhinitis in many parts of the world, has been shown to improve asthma and allergic rhinitis in patients with both these conditions [38]. The study enrolled adult patients with asthma inadequately controlled by inhaled corticosteroids; adding montelukast to the therapy was compared with doubling the inhaled budesonide dose [39]. In the group of patients receiving the doubled budesonide dose, patients with coexisting rhinitis did less well than those without rhinitis; whereas in the montelukast therapy group, patients with coexisting rhinitis performed as well as those without rhinitis [35]. These results suggest that improvements in rhinitis produced by montelukast therapy may be associated with improvements in asthma.

Cigarette smoking is another factor that can adversely affect asthma control. The prevalence of active smoking among adults with asthma is similar to that among those without asthma, tending to be about 25% in developed countries [40]. Cigarette smoking, both active and passive, can increase susceptibility to developing asthma in predisposed individuals [10,40,41]. Moreover, for patients with asthma, active smoking is associated with more severe asthma symptoms and a more rapid decline in lung function than for nonsmokers. Of note, active smokers with asthma tend to be resistant to both oral and inhaled corticosteroid therapy [40]. Smoking cessation is therefore an important component of asthma management. Recent evidence indicates that lung function improves and sputum neutrophil counts fall within several weeks among patients who quit smoking relative to those who do not quit [42].

Adherence to therapy and compliance with physician recommendations are other important components of successful asthma management. Many factors contribute to level of adherence to controller therapy for asthma, including beliefs about the benefits of treatment, concerns about potential adverse effects of treatment, perceived asthma severity, and duration of asthma [43,44]. In addition, cultural factors may influence beliefs about medications [45]. Reported factors influencing adherence among adolescents also include cognitive difficulties, lack of social support, lack of self-efficacy, denial or distrust, and peer and family issues [46].

A complete understanding of the goals of asthma therapy could improve patient adherence to prescribed therapy. Both physicians and patients often overestimate asthma control relative to guideline definitions of control; moreover, patients tend to accept their asthma symptoms and may consider their asthma better controlled than their physicians do [3,4,47]. A better awareness of guideline definitions should therefore be promoted: patients and their families need to understand the nature of asthma symptoms and, most importantly, the criteria defining asthma control.

Finally, as a better understanding is gained of the genetic factors controlling asthma phenotypes, this information should be included in asthma guidelines. Genetic factors are important in determining responders and nonresponders to specific treatments for asthma. For example, patients with aspirin-intolerant asthma show upregulation of leukotriene C_4 _synthase and excess leukotriene production, with responsiveness to leukotriene receptor antagonist therapy [48,49]. A single nucleotide polymorphism (allelic variant C of leukotriene C_4 _synthase) has been identified that shows moderate association with aspirin-intolerant asthma [48]. Polymorphisms of the gene encoding the β_2_-adrenergic receptor (*ADRB2*) appear to play a role in bronchodilator responsiveness and constitute an area of active research [50], the results of which should be reflected in future guidelines.

## Practical tools to assess symptoms and asthma control

Simple questionnaires to assess the presence of asthma and rhinitis symptoms have been proposed in the new International Primary Care Respiratory Group guidelines for the diagnosis of respiratory diseases in primary care [17] (displayed in Tables 3 and 4). Moreover, tools to readily estimate the impairment and impact on quality of life associated with allergic rhinitis and asthma would help healthcare providers to individualize therapy. Many patients consulting general practitioners for allergic rhinitis have substantial impairment in quality of life, sleep, daily activities, and work performance [51].

**Table 3:** Adult asthma questionnaire

1. Have you had wheezing or whistling in your chest at any time in the last 12 months?
2. Have you been woken up at night by an attack of coughing at any time in the last 12 months?
3. Have you been woken up at night by an attack of shortness of breath at any time in the last 12 months?
4. Have you woken up with a feeling of tightness in your chest at any time in the last 12 months?
*If new or recurrent asthma or rhinitis is suspected, assess possible occupational association*.
5. Do your symptoms occur less frequently or not at all on days away from work and on vacations?
*If yes, ask about the nature of the patient's occupation and consider 4-hourly peak flow recording at and away from work and consider referring patient to a specialist for further assessment*.

NOTE: Any patient diagnosed with asthma should also be evaluated for allergic rhinitis.

Reprinted with permission from Levy and coworkers [17].

**Table 4:** Allergic rhinitis questionnaire

1. Do you have any of the following symptoms:*
•Symptoms on only one side of your nose
•Nasal obstruction without other symptoms
•Thick, green or yellow discharge from your nose
•Postnasal drip (down the back of your throat) with thick mucus and/or no runny nose
•Facial pain
•Recurrent nosebleeds
•Inability to smell
2. Do you have any of the following symptoms for at least 1 hour on most days (or on most days during the season if your symptoms are seasonal)?†
•Watery runny nose
•Sneezing, especially violent and in bouts
•Nasal obstruction
•Nasal itching
•Conjunctivitis (red, itchy eyes)

*These symptoms are usually NOT found in allergic rhinitis and the presence of ANY ONE of them suggests that alternative diagnoses should be investigated.

†*Scoring System: The presence of watery runny nose with ONE OR MORE of the other symptoms in this list suggests allergic rhinitis, and indicates that the patient should undergo further diagnostic assessment*.

NOTE: Any patient diagnosed with allergic rhinitis should also be evaluated for asthma.

Reprinted with permission from Levy and coworkers [17].

A practical tool to assess asthma control, the 6-Point Asthma and Allergic Rhinitis Status Measure, has been developed by the General Practice Airways Group with Allergy UK, and is available for download from the General Practice Airways Group website [52]. This short survey includes questions about smoking and the presence of allergic rhinitis symptoms. Suggestions of possible remedies to improve asthma control, as guided by questionnaire results, are provided for the physician. Moreover, assessment of the actual use of prescribed controller medication (adherence) and assessment of inhaler techniques are noted as important evaluations guiding the decision-making process.

## Adherence to guidelines by physicians in practice

Guideline recommendations are only as successful as are the human individuals – the healthcare providers and the patients – in applying them in clinical practice. Primary care physicians often do not follow guidelines [47,53-55]. There is some evidence that general practitioners are not convinced of the applicability of guidelines to their practice settings and that they perceive a tension between primary and secondary care [55]. Guidelines for use in primary care are often written by specialists. Reported barriers to guideline use in primary care vary according to the setting, and include lack of awareness, lack of familiarity, or lack of agreement, as well as lack of self-efficacy, lack of outcome expectancy, inertia of previous practice, and time limitations; external barriers related to guideline factors, patient factors, and environmental factors are also reported [53,56]. For guidelines to be effective, they clearly must be accessible and perceived by the primary care community as relevant to daily clinical practice.

## Conclusion

Practice guidelines are more likely to be followed if they are simple and flexible [57]. The development of country-specific guidelines or, ideally, local guidelines for each region would provide more practical solutions for asthma care and would account for factors, such as social factors and costs, that influence patient choice and adherence to therapy. Pragmatic clinical trials and well-designed observational trials are needed to provide information on the effectiveness of therapies in real-world settings. Finally, practical tools to facilitate the diagnosis and assessment of asthma and factors responsible for poor control, such as associated allergic rhinitis, limited adherence, and smoking behavior, are needed to supplement treatment information provided in clinical practice guidelines for asthma.

## Abbreviations

ARIA = Allergic Rhinitis and its Impact on Asthma; GINA = Global Initiative for Asthma; RCT = randomized controlled trial.

## Competing interests

DP has received honoraria for speaking at sponsored meetings from the following companies marketing respiratory products: 3 M, Altana, AstraZeneca, Boehringer Ingelheim, GlaxoSmithKline, IVAX, Merck Sharp & Dohme, Novartis, Pfizer and Schering-Plough. DP has also received honoraria for advisory panels with 3 M, Altana, AstraZeneca, Boehringer Ingelheim, GlaxoSmithKline, IVAX, MSD, Novartis, Pfizer and Schering-Plough. DP or his research team have received funding for research projects from 3 M, Altana, AstraZeneca, Boehringer Ingelheim, GlaxoSmithKline, IVAX, Merck, Sharp & Dohme, Novartis, Pfizer, Schering-Plough, Viatris.

MT has no shares in any pharmaceutical company. Either through his role at the University of Aberdeen or personally MT has received grants, honoraria or educational support from the following companies as well as the UK NHS R&D programme and Asthma UK: Altana, AstraZeneca, GlaxoSmithKline, Ivax, Merck, Sharp and Dohme, Novartis, Schering Plough, Trinity Pharmaceuticals, Viatris.
